# Male Gender is independently associated with pulmonary tuberculosis among sputum and non-sputum producers people with presumptive tuberculosis in Southwestern Uganda

**DOI:** 10.1186/s12879-014-0638-5

**Published:** 2014-12-10

**Authors:** Yap Boum, Daniel Atwine, Patrick Orikiriza, Justus Assimwe, Anne-Laure Page, Juliet Mwanga-Amumpaire, Maryline Bonnet

**Affiliations:** Epicentre Mbarara, Mbarara, Uganda; Epicentre, Paris, France; Mbarara University of Science and Technology, Mbarara, Uganda; Uganda National Tuberculosis and Leprosy program, Mbarara, Uganda

**Keywords:** Sex, HIV, Risk factors, String test, Sputum induction

## Abstract

**Background:**

Little is known about the association between gender and risk of TB infection. We sought to assess the impact of gender on TB prevalence among people with presumptive tuberculosis at a regional referral hospital in a high TB and HIV prevalence setting.

**Methods:**

We analyzed data from two diagnostic TB studies conducted in rural, southwestern Uganda. People with presumptive tuberculosis were evaluated by chest X-ray, fluorescence microscopy, TB culture, and HIV testing. Our primary outcome of interest was TB infection, as defined by a positive TB culture. Our primary explanatory variable of interest was gender. We fit univariable and multivariable logistic regression models to investigate associations between TB infection and gender, before and after adjusting or possible confounding factors, including ability to produce sputum, age and residence.

**Results:**

Between April 2010 and September 2012, 863 people with presumptive tuberculosis (PWPTB) were enrolled in the two studies at Mbarara Regional Referral Hospital (MRRH) in Uganda. Among them 664 (76.9%) were able to produce sputum. X-ray was suggestive of TB for 258 (66.5%) of males and 175 (44.8%) of female (p < 0.001). using microscopy 84 (20%) of males and 48 (10.9%) of females were diagnosed with TB (p < 0.001) while 122 (30.3%) of males and 76 (18.4%) of females were diagnosed with TB (p < 0.001) using TB culture.

In multivariable logistic regression models, the odds of having TB was higher in males than females (AOR 2.2 (1.56-3.18 95% CI°, P < 0.001), after adjustment for age, HIV status, ability to produce sputum, and residence.

**Conclusion:**

In Southwestern Uganda, TB prevalence is higher among male than female people with presumptive TB. The increased risk of TB among males is independent of other TB risk factors. These findings emphasize the need for gender-focused interventions aimed at reducing TB transmission.

**Electronic supplementary material:**

The online version of this article (doi:10.1186/s12879-014-0638-5) contains supplementary material, which is available to authorized users.

## Background

The World Health Organization (WHO) stated that the global male to female prevalence ratio of tuberculosis (TB) is 1.85 [1]. This gender imbalance increases with age and may have implications in the management of TB [1].While the causes of gender imbalance in TB diagnosis remain unclear and vary among countries, most published studies on the association between gender and TB have not assessed different possible contributing factors in the same setting. To our knowledge, no studies have examined gender differences in non-sputum-producers, mainly HIV infected patients, to assess the effect of gender on TB diagnosis in this population. Moreover, there are no published data on TB prevalence by gender in Southwestern Uganda, a high TB and HIV prevalence setting [2],[3].

We determined the prevalence of culture confirmed tuberculosis among men and women presenting to a regional referral hospital in southwestern Ugandan with presumptive tuberculosis (PWPTB) and we assessed the effect of gender on the diagnosis of TB.

## Methods

### Study design

We conducted a secondary analysis of data collected in two cross-sectional diagnostic studies: 1) a study that evaluated the performance of colorimetric culture methods for diagnosis of tuberculosis [4] (“colorimetric study”) and 2) a study that assessed the detection yield of alternative specimen collection methods (the “string test study”) in patients unable to produce sputum [5].

### Participants

Both studies consecutively recruited participants from the outpatient department (OPD) and the HIV clinic (Immune Suppression Syndrome [ISS] Clinic) at the Mbarara Regional Referral Hospital (MRRH). Patients were eligible if they reported a cough lasting more than two weeks and were older than 15 years of age. Patients able to produce at least 1 mL of sputum were enrolled in the colorimetric study and patients with less than 1 mL of sputum or who were unable to produce sputum spontaneously were included in the string test study. In addition, patients without cough but with unexplained weight loss, fever or recent chest x-ray showing radiological features compatible with PTB and who could not produce sputum were eligible for participation in the string test study. Participants were excluded if they had more than seven days of anti-TB treatment prior to enrollment and/or if they were too sick to comply with the sputum induction procedure of the string test study.

### Study procedures

Socio-demographic characteristics specifically age, gender and residence, were collected using a standardized questionnaire which was administered in the local language. Participants underwent a physical examination by a clinical officer to record data on duration and nature of signs and symptoms. Participants were tested for HIV using the algorithm recommended by the Ugandan Ministry of Health STD/AIDS Control Program [4]. Finally, all participants had an antero-posterior chest X-ray. Chest x-ray images were interpreted by study clinicians according to a predetermined pictorial tick sheet with quality control by the hospital radiologist and reported as: “normal”, “abnormal, possible TB”, or “highly suggestive of TB”. For this analysis, we considered the latter as radiographic evidence of TB.

For patients enrolled in the colorimetric study [4], two sputum samples were collected including one at the time of enrollment and a second the following morning. For patients enrolled in the string test study [5], participants swallowed a weighted gelatin capsule filled with 90 cm of nylon string (Entero-test®, HDC Corporation, San Jose, California, USA), which was retrieved after 2 hours followed by a sputum induction using 5% hypertonic saline solution nebulized for a maximum of 20 minutes. Two samples were collected during two consecutive days.

Each collected specimen was processed by auramine LED-fluorescence microscopy (on fresh sputum and sediment sample after string test and sputum induction) [4] and TB culture, using both Lowenstein-Jensen (LJ) tubes and a manual mycobacterium growth indicator tube (MGIT) (Becton Dickinson, Franklin Lakes, New Jersey, USA) [4],[5]. Observation of acid-fast bacilli (AFB) under the fluorescent microscope was considered a positive smear result, and observation of growth suggestive of TB on/in TB selective media and confirmed by biochemical/serological tests was considered a positive culture result.

### Statistical analysis

Patient,’ specimen characteristics, and test results were summarized by gender, and compared using chi-square test for categorical variables and t-test or rank-sum non-parametric tests for continuous variables. We defined our primary outcome of interest as a culture diagnosis of TB. Our primary explanatory variable of interest was gender. Secondary predictors included age, HIV serostatus, productive versus non-productive cough, urban versus rural residence, presence or absence of a chest x-ray highly suggestive of TB, sputum volume (greater than or less than 1 mL [6], and macroscopic specimen appearance (mucopurulent/purulent or mucoid/mucosalivary/salivary/bloody versus not). We fitted univariable and multivariable logistic regression models to assess for relationships between gender and our outcome of interest, before and after adjustment for confounding variables.

Data was analyzed with STATA, version 11 (StataCorp, College Station, Texas, USA). A confidence level of 95% was deemed as statistically significant.

### Ethical approval

Both study protocols were approved by the CPP Ile de France, the Mbarara University Faculty of Medicine Research and Ethics Committee and Institutional Review Committee and the Uganda National Council for Science and Technology. All participants gave written informed consent.

## Results

### Participant’s enrollment and characteristics

**Figure 1:**
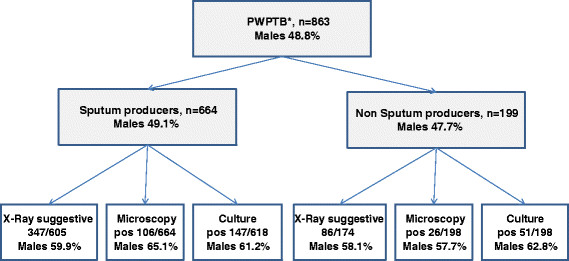
Study profile (*PWPTB patient with presumptive tuberculosis). Number of patients enrolled in the study, results by diagnosis tools with the proportion of positive results among the general population and the percentage of males in the patients with a positive result.

A total of 863 participants were enrolled in the two studies April 2010 and September 2012, and were included in this analyses. 664 participants (76.9%) were enrolled in the colorimetric study and 199 (23.1%) in the string study (Figure 1).

We enrolled 441 (51%) female participants. Female PWPTB were younger than males, had a higher prevalence of HIV sero-positivity and were less likely to have chest X-ray results suggestive of pulmonary TB than males (Table 1). There were no differences in other socio-demographic or clinical characteristics by gender.

**Table 1:** **Patient characteristics and by gender**

Variables	Total n (%)	Male n (%)	Female n (%)	P value
**Study**	863 (100.0)	421 (48.8)	442 (51.2)	0.737
**Sputum producers**	664 (76.9)	326 (49.1)	338 (50.9)	
**Non sputum producers**	199 (23.1)	95 (47.7)	104 (52.3)	
**Age in years (N = 852)**	mean (SD)	mean (SD)	mean (SD)	
	39.7 (13.9)	41.2 (14.3)	38.2 (13.4)	**0.001**
**Age categories in years (N = 852)**	**Total n (%)**	**Male n (%)**	**Female n (%)**	**0.005**
<25	102 (12.0)	49 (48.0)	53 (52.0)	
25-35	269 (31.6)	113 (42.0)	156 (58.0)	
36-45	241 (28.3)	119 (49.4)	122 (50.6)	
>45	240 (28.2)	139 (57.9)	101 (42.1)	
**HIV positive, N = 820 (%)**				**<0.001**
Negative	345 (42.1)	196 (56.8)	149 (43.2)	
Positive	475 (57.9)	207 (43.6)	268 (56.4)	
**Residence (N = 860)**				0.411
Urban	427 (49.6)	203 (47.5)	224 (52.5)	
Rural	433 (50.4)	218 (50.6)	215 (49.4)	
**X-ray ,N = 779 (%)**				**<0.001**
Suggestive of TB	433 (55.6)	258 (66.5)	175 (44.8)	
Non suggestive of TB	346 (44.4)	130 (33.5)	216 (55.2)	

### Specimens’ characteristics, microscopy and culture results

Sputum specimen characteristics were similar between males and females (Table 1). Significantly more males than females had positive TB microscopy (84/421 [20%] versus 48/441 [11%], *P* < 0.001, Table 2).

**Table 2:** **Specimens’ characteristics and laboratory results by gender and study**

Colorimetric study (sputum producers)	Total n (%)	Male n (%)	Female n (%)	p
Sputum volume (n = 689) More than 1 ml	672 (97.5)	331 (97.6)	341 (97.4)	0.858
Sputum Quality (n = 681) Purulent-mucopurulent	223 (32.7)	117 (35.2)	106 (30.4)	0.176
Microscopy (N = 664) Positive	106 (16.0)	69 (21.2)	37 (10.9)	<0.001
TB Culture (N = 618) Positive	147 (23.8)	90 (29.3)	57 (18.3)	0.001
Xray (N = 605) suggestive	347 (57.4)	208 (68.4)	139 (46.2)	< 0.001
**String test and sputum induction study (non sputum producers)**	**Total n (%)**	**Male n (%)**	**Female n (%)**	**p**
Microscopy (N = 198) Positive	26 (13.1)	15 (15.8)	11 (10.7)	0.288
TB Culture (N = 198) Positive	51 (25.7)	32 (33.7)	19 (18.5)	0.014
Xray (N = 174) suggestive	86 (49.4)	50 (59.5)	36 (40.0)	0.010
**Both studies**	**Total n (%)**	**Male n (%)**	**Female n (%)**	**p**
Microscopy (N = 862) Positive	132 (15.3)	84 (20.0)	48 (10.9)	< 0.001
TB Culture (N = 816) Positive	198 (24.3)	122 (30.3)	76 (18.4)	< 0.001
Xray (N = 779) suggestive	433 (55.6)	258 (66.5)	175 (44.8)	< 0.001

### Outcomes

**Figure 2:**
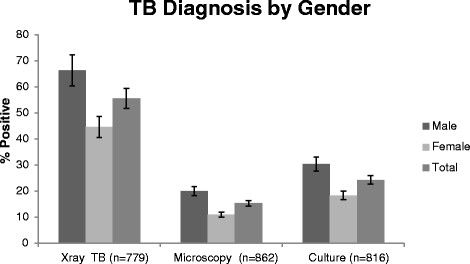
**Proportion of X-ray suggestive of TB and TB positive results by microscopy and culture stratified by gender.** The right column represents the proportion of male, the middle column represent the proportion of female and the left column the proportion of both in the general population.

**Figure 3:**
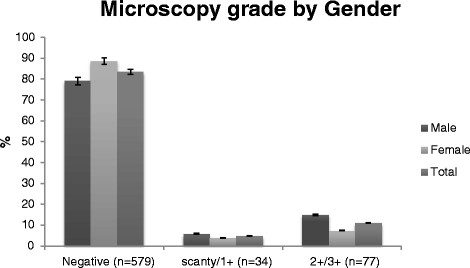
**Proportions of microscopy results by gender in sputum producers with presumptive TB.** The right column represents the proportion of male, the middle column represent the proportion of female and the left column the proportion of both in the general population.

Males were more likely to have a positive AFB sputum smear, and more likely to have high grade (2+/3+) smear results (Table 2, Figures 2 and 3). Males were also more likely to have a positive TB culture (30.3% versus 18.4%, *P* = 0.001, Table 2). This was true both for those who produced sputum and those who could not produce sputum. Of the participants with smear-negative results, the males were more likely to have culture positive TB (males: 40/318, 12.6%, versus females: 29/367, 7.9%; *P* = 0.043).

In univariable models gender, age, X-ray and microscopy were associated with a diagnosis of TB by culture. In multivariable models, gender remained significant (AOR 2.2 95%CI [1.56-3.18]). We also found independent associations with a TB diagnosis for age (Table 3).

**Table 3:** **Unadjusted and adjusted odds ratios for the relationship between gender and TB culture positivity (N = 779)**

Characteristics	OR (95% CI)	P value	AOR (95% CI)	P value
**Gender**		**<0.001**		**<0.001**
Female	REF		REF	
Male	1.9 (1.40-2.69)		2.2 (1.56-3.18)	
**Age (categories in years)**		**<0.001**		**<0.001**
>45	REF		REF	
36-45	1.8 (1.09-2.85)		1.88 (1.13-3.12)	
25-35	2.2 (1.40-3.52)		2.5 (1.56-4.15)	
<25	3.3 (1.90-5.71)		3.4 (1.90-6.11)	
**X-Ray**				
Non Suggestive of TB	REF			
Suggestive of TB	5.0 (3.29-7.57)	**0.001**		
**Microscopy**				
Negative	REF			
Positive	575 (139.3-2378.9)	**<0.001**		
**Type of cough**		0.5751		
Non-productive	REF			
Productive	1.0 (0.77-1.61)			
**HIV status**		0.1914		
Negative	REF			
Positive	0.8 (0.57-1.12)			
**Residence**		0.977		
Urban	REF			
Rural	1.0 (0.73-1.38)			

## Discussion

In a prospective cohort of people with presumptive tuberculosis at a regional hospital in rural Uganda, male people with presumptive tuberculosis were twice as likely to be diagnosed with culture positive TB compared to females. Although the proportion of males and females seeking care was similar, males represented the majority of culture confirmed TB patients irrespective of their ability to produce sputum, the sputum volume and quality and the HIV status.

Our data are in agreement with data from other settings, which also demonstrate a higher proportion of males with TB culture confirmed compared to women and a higher prevalence of TB in young women [7]-[11].

TB prevalence in males versus females varies widely among countries, and in some areas prevalence is higher among women. In Pakistan, the majority of TB patients across all age groups are females [12], and in Peru a higher prevalence of TB was found in women of reproductive age compared to males in the same age range [13].These countries are an exception, however. In Africa and Eastern Europe males seem to represent the majority of adult patients with TB [14]-[16]. In fact the figures are higher in Eastern Europe than African countries with prevalence as high as 66% and 88% in Uzbekistan and Belarus respectively [7],[8].

Various reasons have been suggested to explain this gender imbalance in TB prevalence. Less access to health care for women, and therefore more unreported TB, has been mentioned in many countries [2],[15],[17], and the potentially less sensitive screening and diagnosis strategies for women [15] has led to underestimation of TB in women [15]. Another contributing factor suggested is the higher prevalence of HIV among women in many countries. The paucibacillary nature of pulmonary TB that is common in high HIV prevalence settings leads to an increased proportion of microscopy smear negative results. Therefore any gender disparities in HIV prevalence setting are likely to be reflected in TB detection because of the association of HIV with a reduced performance of TB diagnostic tests, especially microscopy [18]. Reliance on microscopy for diagnosing TB in most resource-limited settings could underestimate the TB burden in women who are more likely to be HIV infected than men and typically produce a lower quality of sputum specimen [18].Other explanations such as social behaviors among men and the difference between male and female susceptibility to TB have also been mentioned [19],[20].

It is unlikely that test characteristics could explain the difference we found in TB detection by gender. We used highly sensitive methods for diagnosing pulmonary TB based on the combination of solid and liquid culture methods [21] and on the use of alternative specimen collection methods when patients were unable to produce sputum. Therefore, it is very unlikely that the difference of prevalence of TB between men and women is due to under TB diagnosis in women. The use of culture increased the detection of TB as described by Lawson et *al*. in Nigeria [16], who reported similar performance of culture among males and females [16]. In agreement with previous studies women tend to have less smear-positive pulmonary TB than men. The association between the quality of sputum specimen and the performance of smear-microscopy is well known and could explain partially the gender imbalance among smear positive TB patients, because men tend to produce a better sputum than women [18]. However, in contrast to other studies, in our study there was no difference in volume or quality of sputum specimen between men and women. Moreover the proportion of men having TB remains significantly higher than females in non-sputum producers (33.7% Vs 18.4% respectively, p = 0.014).

It is likely that differences in health seeking behaviors are at least partially responsible for the gender-based difference in TB detection we demonstrated. While we did not find a difference in district of residence (as a surrogate of distance to health center), many factors can explain differences in access to health care between men and women, including stigmatization and social factors were not assessed in our study. It has been observed in several settings that women have better health-seeking behaviors compared to men and that men have more advanced symptoms by the time they seek healthcare [22],[23]. Similar differences in HIV serostatus, presentation to HIV care, HIV-related mortality have been found by gender [24]. This is likely to be the same in our setting. Indeed, the bacterial load and the proportion of suggestive chest X-ray were significantly higher among men than women, which suggests a late stage of the disease most likely due to late presentation for health care services [18]. Moreover males represent 85% of patients admitted in the TB ward of Mbarara Hospital, where patients are admitted at a late stage of the disease and require higher level of care, hence confirming our finding (Unpublished data).

Unexpectedly, HIV infection was not associated with TB culture positive results, yet TB is more prevalent among HIV-positive people [8] and MTB detection is known to be less sensitive in HIV infected patients than the HIV non infected [19]. The association between gender and culture results was the same in HIV infected and non-infected patients. Similar findings were described by Lawson *et al*. in Nigeria [16] with smear microscopy among women. However, in their study, HIV-infected males were less likely to have positive smears than HIV-negative males.

The study has some limitations. First, it focused on patients referred to a regional referral hospital, which might not reflect the same population of people who access health care in rural areas, and might reflect care for those who have been referred on after failure of local therapy or diagnostics. Indeed, an important proportion of the population of Southwestern Uganda does not reach the regional hospital and seeks care in peripheral health centers. Therefore, a higher proportion of women with TB could be potentially diagnosed in more remote facilities. Nevertheless, data from all health centers of the region suggest that this is not the case: males represented 65% of the 4438 TB cases detected in the Southwestern region during the period of our study, which is similar to the proportion of male among culture positive patients in the study (61.6%). (Asiimwe Justus, personal communication). Secondly, the study did not include any information to assess health seeking behavior and access to health services between men and women as potential factors that could explain differences we found.

## Conclusion

In Southwestern Uganda, we found significantly higher odds of a diagnosis of TB among male people with presumptive tuberculosis. This difference was independent of age, HIV-serostatus, and residence. The higher prevalence of TB in men attending the TB clinic is not due to under-diagnosis in women and seems to be related to delayed diagnosis of TB in men, most likely due to late presentation. These findings emphasize the need for research on understanding social and potential biological determinants of higher prevalence of TB in men and to identify the barriers faced by men in seeking TB healthcare in the early stage of TB. Gender based interventions, such as TB health education targeting men or active screening of TB in men should be pursued. Our data also highlight the need for more targeted interventions in young women, who are at high risk of transmitting TB to children and, re-emphasize the need for TB contact tracing.

## Authors’ original submitted files for images

Below are the links to the authors’ original submitted files for images. Authors’ original file for figure 1 Authors’ original file for figure 2 Authors’ original file for figure 3
