# Supplementary figures and images for: Male Gender is independently associated with pulmonary tuberculosis among sputum and non-sputum producers people with presumptive tuberculosis in Southwestern Uganda
Source: BMC Infect Dis. 2014 Dec 10;14:638. doi: 10.1186/s12879-014-0638-5 (PMC4265338; doi:10.1186/s12879-014-0638-5)

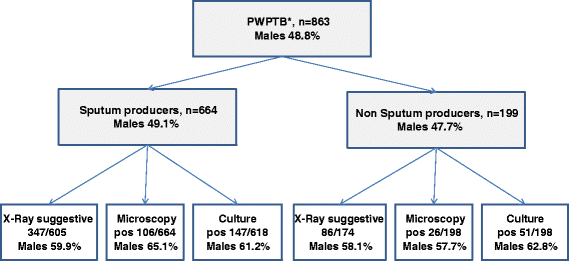

Supplement: Supplementary file 1 — Authors’ original file for figure 1 [file 12879_2014_638_MOESM1_ESM.gif]

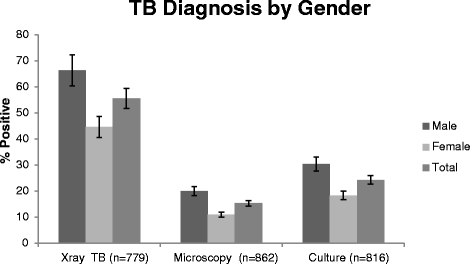

Supplement: Supplementary file 2 — Authors’ original file for figure 2 [file 12879_2014_638_MOESM2_ESM.gif]

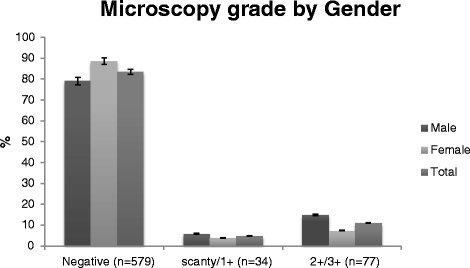

Supplement: Supplementary file 3 — Authors’ original file for figure 3 [file 12879_2014_638_MOESM3_ESM.gif]
